# Estimation of the population size of gay, bisexual and other men who have sex with men in Canada, 2020

**DOI:** 10.14745/ccdr.v49i1112a02

**Published:** 2023-11-01

**Authors:** Justin Sorge, Sean Colyer, Joseph Cox, Abigail Kroch, Nathan Lachowsky, Nashira Popovic, Qiuying Yang

**Affiliations:** 1 Public Health Agency of Canada, Ottawa, ON; 2 Ontario HIV Treatment Network, Toronto, ON; 3 Public Health Ontario, Toronto, ON; 4 Dalla Lana School of Public Health, University of Toronto, Toronto, ON; 5 Community-Based Research Centre, Vancouver, BC; 6 School of Public Health & Social Policy, University of Victoria, Victoria, BC

**Keywords:** population size estimation, HIV, gay bisexual and other men who have sex with men, key populations

## Abstract

**Background:**

Gay or bisexual (GB) and other men who have sex with men (MSM) are disproportionately affected by human immunodeficiency virus (HIV) globally and domestically in Canada. Reliable and recent population size estimates are necessary to allocate resources to meet prevention needs and for modelling the HIV epidemic. However, previous direct estimates did not account for GB men who would not reveal their sexual identity to a government survey, nor MSM not identifying as GB. The objective of this study was to develop two national population size estimates of gay, bisexual and other men who have sex with men (gbMSM) in 2020. First, GB men based on identity, regardless of sexual experience, and MSM who do not identify as GB but reported anal sex with a man in the past 1–5 years ("Identity-or-Behaviour” estimate). Second, an estimate of gbMSM who reported past 6–12 months anal sex with a man ("Behaviour-only” estimate).

**Methods:**

Estimates for males aged 15 years and older were drawn from Statistics Canada’s population size estimates, the Canadian Community Health Survey and the Community-Based Research Centre’s Sex Now Survey. Estimated proportions of GB identity, those not likely to disclose GB identity and MSM who do not identify as GB but who reported past 1–5 years anal sex were applied. Past 6–12 months anal sex history was subsequently used to limit estimates to those sexually active anally.

**Results:**

It was estimated that 3.5% of the male population in Canada aged 15 years and older identified as GB. Of GB males, 86.5% were likely to disclose their sexual identity to a government survey. A further 0.1% of non-GB identified males reported past year anal sex with a man. The national Identity-or-Behaviour gbMSM population size in 2020 was estimated at 669,613 people, equivalent to 4.3% of the Canadian male population aged 15 years and older. The estimate of Behaviour-only gbMSM was 412,186, representing 2.6% of the Canadian male population aged 15 years and older.

**Conclusion:**

Using data from multiple sources, a model applied to estimate the population size of gbMSM, accounting for populations previously not included in prior estimates, has been described.

## Introduction

Nationally and globally, men who identify as gay or bisexual (GB) and other men who have sex with men (MSM) are overrepresented among people living with the human immunodeficiency virus (HIV) ((1,2)). Gay, bisexual and other men who have sex with men (gbMSM) account for approximately half of new HIV infections in Canada, despite representing only 2%–4% of adult males ((2)). Reliable population size estimates are necessary to inform resource allocation to meet prevention, testing and treatment needs and for modelling the HIV epidemic ((3)).

A nuanced understanding of the HIV epidemic among gbMSM has been limited by the challenges estimating this population size: lack of population sampling frame ((4)); small sample sizes in general population health surveys; stigma surrounding sexual orientation disclosure on health surveys ((5,6)); and inconsistent measurement of sexual orientation ((7)). The previous Canadian national estimate of 349,837 people (representing 2.4% of the male population aged 15 years and older), was published in 2014 and used for calculating population-specific estimates of HIV burden. This estimate was derived using direct estimation of self-identifying GB men who reported having sex with a man in the past 12 months on a population health survey. This estimate did not account for GB-identifying men who did not disclose their sexual orientation on a government survey, non-GB-identifying MSM or GB-identifying men who had not reached sexual debut ((8)). As such, previous estimates of this population size at national and subnational levels needed to be updated and made more comprehensive. Several other methods have been employed to estimate the population size at the local level. The “Wisdom of the Crowd” (WotC) method is based on the perceived size of the population by a sample of community members. The multiplier method estimates population size by triangulating information on group membership (e.g. HIV testers) with the proportion that report being a member of that group on a population survey. Successive/respondent-driven sampling methods utilize a Bayesian probability model and incorporate information on the sampling process. Mapping techniques enumerate community members in places where they congregate ((9–11)).

The primary objective of this study was to estimate the gbMSM population size in 10 provinces in Canada as of 2020. As gbMSM are both an identity and behavioural-based community, the aim was to calculate population size estimates based on three dimensions: 1) GB identity (regardless of sexual experience) 2) MSM who do not identify as GB but reported anal sex with a man in the past 1–5 years and, 3) an estimate of gbMSM reporting past 6–12 months anal sex with a man.

A secondary objective was to provide population size estimates 1) by region and 2) by rural areas and small population centres (small population areas defined as population size fewer than 30,000) versus medium and large urban population centres (large population areas defined as population size 30,000 or more).

## Methods

### Definitions

In an effort to be inclusive of all members of the population, efforts were taken to produce estimates incorporating both self-identity and recent anal intercourse as a risk for transmission of HIV and other sexually transmitted and blood-borne infections (STBBIs). Population size estimates of gbMSM were calculated for the following groups: 1) Identity-or-Behaviour—GB-identifying men, regardless of anal sex experience (identity), plus men who do not identify as GB but reported anal sex with a man in the past 1–5 years (behaviour); and 2) Behaviour-only—GB-identifying men who reported anal sex with a man in the past 6–12 months, plus men who do not identify as GB but who reported anal sex with a man in the past 6–12 months. Note, differing time frames (1 or 5 years and 6 or 12 months) is dependant on data source used, see below.

The Behaviour-only estimate was directly applicable to the development of policy, allocation of resources and modelling of diseases transmitted through anal sex, such as HIV and other STBBIs. An Identity-or-Behaviour estimate was derived that is inclusive of MSM community members who were previously excluded from estimation, regardless of sexual orientation. It was important to include MSM based on both anal sex experience and GB identity 1) for representativeness, 2) because it may be useful in primary prevention and health promotion efforts beyond sexual health and 3) in the context of sexual networks and potential bridges to populations outside existing gbMSM networks.

### Data sources

**Canadian Community Health Survey:** The Canadian Community Health Survey (CCHS) is a national cross-sectional population survey of health status, healthcare utilization and health determinants. The CCHS, through a multistage probability sample allocation strategy, attempts to create a nationally representative sample of Canadians. Altogether, the CCHS sampling strategy covers 97% of Canadians aged 12 years and older ((12,13)). The CCHS collects data from the Canadian population via an interviewer-administered electronic questionnaire or computer-assisted telephone interview. The 2019–2020 cycles asked participants, “What is your sexual orientation?” Response options “heterosexual”, “gay or lesbian”, “bisexual” and “sexual orientation not elsewhere classified” dichotomized as “gay/bisexual” and “not elsewhere specified”. Participants were also asked a separate, unlinked question: “In the past 12 months, have you had sex with a male?”, which is defined in CCHS as “vaginal or anal” (yes/no). Statistics Canada provides users with bootstrap weights to estimate the sampling variance. The two cycles of CCHS data were combined as per the description by Thomas and Wannell ((14)). The CCHS analyses were restricted to male participants aged 15 years and older.

**Sex Now Survey:** Sex Now is Canada’s largest community-based health survey specifically targeting Two Spirit, gay, bisexual, transgender and queer (2SGBTQ) men ((15,16)). Multiple recruitment methods were utilized to ensure the inclusion of a diverse pool of participants. Promotional material was shared through social media ad buys, prominent drag queen promotion, and ad buys on popular sex-seeking apps (Grindr, Squirt, Scruff, Jack’d), porn sites (PornHub) and 2SGBTQ-oriented media sites. Consenting participants were aged 15 years and older, lived in Canada, were capable of completing the survey in French or English, self-identified as a man or gender other than a woman, and identified as non-heterosexual or report sex with a man in the past five years. During November 2019 and February 2020, through an internet-based, self-administered survey, participants were asked, “How do you identify sexually?” Responses dichotomized as “gay/bisexual” and “other”, which included “asexual”, “straight”, “pansexual”, “queer” and “heteroflexible” identities. Past six-month anal sex experience among participants was based on non-zero responses to the following questions: 1) “In the PAST SIX MONTHS how many men have you had sex with?” and 2) “Of those, how many have you had ANAL sex with IN THE PAST SIX MONTHS?” The first question was used to quantify the denominator of respondents who received the nested question specifically about anal sex experience to calculate the proportion. The survey also asked, “How likely or unlikely would you be to reveal [your sexual orientation], if asked in a Statistics Canada survey (e.g., Census, [CCHS])?” Response options “very likely”, “likely”, “unlikely” and “very unlikely” dichotomized as “likely” and “unlikely.”

### Population size estimate

Statistics Canada provided custom postcensal Canadian 2020 population estimates for males aged 15 years and older for each province and stratified into small and large population areas. To estimate the Identity-or-Behaviour group, the proportion reporting GB-self-identity from CCHS was applied to the total male aged 15 years and older population count. This number was adjusted to also include those who would be unlikely to disclose GB identity on a Statistics Canada survey per the Sex Now results. To this, estimates of non-GB-identifying MSM were added, which was calculated separately as past 12 months anal sex with a man for CCHS data and past five years anal sex with a man from Sex Now.

To estimate the Behaviour-only group, the process is repeated among respondents reporting recent anal sex with a man (past six months Sex Now/past year CCHS) ((17,18)). Because the CCHS is a general population health survey, while Sex Now is a survey among 2SGBTQ men, analyses were treated differently. We developed separate models, as depicted below in equations 1 to 4:

Equation 1:

**Table:** 

	${IB}_{CCHS}=\left\{{Pop}_{count}*\left[\frac{{GB}_{CCHS\;prop}}{{Disclosure}_{SN\;prop}}\right]\right\}+\left({Pop}_{count}*{MSM}_{CCHS\;prop}\right)$	

IB_CCHS_ = {Pop_count_ * [GB_CCHS prop_ / Disclosure_SN prop_]} + (Pop_count_ * MSM_CCHS prop_)

Where:

*IB_CCHS_* is the Identity-or-Behaviour population size estimate of gbMSM estimated using CCHS data

*Pop_count_* is the count of males aged 15 years and older population

*GB_CCHS prop_* is the proportion of CCHS respondents identifying as GB, relative to the sample

*Disclosure_SN prop_* is the proportion of GB Sex Now respondents likely to report their sexual orientation on a Statistics Canada survey, relative to GB respondents

*MSM_CCHS prop_* is the proportion of CCHS respondents who did not identify as GB and report past year sex with a man, relative to the sample

Equation 2:

**Table:** 

	${IB}_{SN}=\frac{\left\{{Pop}_{count}*\left[\frac{{GB}_{CCHS\;prop}}{{Disclosure}_{SN\;prop}}\right]\right\}}{{GB}_{SN\;prop}}$	

IB_SN_ = {Pop_count_ * [GB_CCHS prop_ / Disclosure_SN prop_]} / GB_SN prop_

Where:

*IB_SN_* is the Identity-or-Behaviour population size estimate of gbMSM estimated using Sex Now data

*Pop_count_* is the count of males aged 15 years and older population

*GB_CCHS prop_* is the proportion of CCHS respondents identifying as GB, relative to the sample

*Disclosure_SN prop_* is the proportion of GB Sex Now respondents likely to report their sexual orientation on a Statistics Canada survey, relative to GB respondents

*GB_SN prop_* is the proportion of Sex Now respondents identifying as GB, relative to the sample

Equation 3:

**Table:** 

	$B_{CCHS}=\left\{{Pop}_{count}*\left[\frac{{GB}_{CCHS\;prop\;p12m\;anal}}{{Disclosure}_{SN\;prop}}\right]\right\}+\left({Pop}_{count}*{MSM}_{CCHS\;prop}\right)$	

B_CCHS_ = {Pop_count_ * [GB_CCHS prop p12m anal_ / Disclosure_SN prop_]} + (Pop_count_ * MSM_CCHS prop_)

Where:

*B_CCHS_* is the Behaviour-only population size estimate of gbMSM estimated using CCHS data

*Pop_count_* is the count of males aged 15 years and older population

*GB_CCHS prop p12m anal_* is the proportion of CCHS respondents identifying as GB and report past year anal sex with a man, relative to the sample

*Disclosure_SN prop_* is the proportion of GB Sex Now respondents likely to report their sexual orientation on a Statistics Canada survey, relative to GB respondents

*MSM_CCHS prop_* is the proportion of CCHS respondents who did not identify as GB and report past year sex with a man, relative to the sample

Equation 4:

**Table:** 

	$B_{SN}=\frac{\left\{{Pop}_{count}*\left[\frac{{GB}_{CCHS\;prop}}{{Disclosure}_{SN\;prop}}\right]\right\}*{GB}_{SN\;prop\;p6m\;anal\;among\;GB}}{{p6m\;Anal}_{SN\;prop\;GB\;among\_all}}$	

B_SN_ = ({Pop_count_ * [GB_CCHS prop_ / Disclosure_SN prop_]} * GB_SN prop p6m anal among GB_) / p6m Anal_SN prop GB among all_

Where:

*B_SN_* is the Behaviour-only population size estimate of gbMSM estimated using CCHS data

*Pop_count_* is the count of males aged 15 years and older population

*GB_CCHS prop_* is the proportion of CCHS respondents identifying as GB, relative to the sample

*Disclosure_SN prop_* is the proportion of GB Sex Now respondents likely to report their sexual orientation on a Statistics Canada survey, relative to GB respondents

*GB_SN prop p6m anal among GB_* is the proportion of Sex Now respondents identifying as GB and reporting past six months anal sex with a man, relative to the GB respondents

*p6m Anal_SN prop_GB_among_all_* is the proportion of Sex Now respondents identifying as GB reporting past six months anal sex with a man, relative to all respondents reporting past six months anal sex with a man

This process was repeated at the regional-level and ‘’small” and “large” population areas (see **Appendix**). Regions were defined as Atlantic (Newfoundland and Labrador, Prince Edward Island, Nova Scotia, New Brunswick), Québec, Ontario, Prairies (Manitoba, Saskatchewan, Alberta) and British Columbia.

### Analysis

Analyses were done in SAS Enterprise Guide version 7.15 ((19)). Weighted estimation and bootstrap variance were used to calculate CCHS model inputs and 95% confidence intervals using the PROC SURVEYFREQ procedure. Sex Now model input 95% confidence intervals were calculated, unweighted, using the PROC FREQ procedure using binomial proportion. For the population size estimates, point estimates were calculated as the mean between CCHS- and Sex Now-derived estimates and upper and lower bounds were taken as Sex Now and CCHS estimates, respectively ((17,18)). Results are presented as counts (range) and percentage (range). Missing data were treated as non-response and excluded from analysis.

## Results

The 2020 estimated male population of all Canadian provinces aged 15 years and older was 15,762,949. Direct weighted estimates of GB self-identity from CCHS totalled 496,594 people (3.5% of the male aged 15 years and older sample). Of 10,541 Sex Now participants beginning the questionnaire, 9,693 (92.0%) respondents self-identified as GB. Among 9,525 remaining GB participants who completed the question, 8,241 (86.5%) reported being likely to disclose their sexual orientation on a Statistics Canada survey. A further 21,380 (0.1% of the CCHS sample) of CCHS respondents who did not self-identify as GB reported anal sex with a man in the past year. In addition, the Sex Now sample included 848 (8.0%) of respondents identifying as a sexual orientation other than GB who reported sex with a man in the past five years.

A total of 218,705 (1.5% of the CCHS sample, 44.0% of GB respondents) self-identifying GB respondents reported anal sex with a man in the past year. Among the 5,791 remaining Sex Now GB participants, 4,561 (78.8%) reported anal sex with a man in the previous six months. While among the 460 remaining respondents identifying as a sexual orientation other than GB, 246 (53.5%) reported anal sex with a man in the previous six months. Among all respondents reporting past six months anal sex, 94.9% self-identified as GB. Note, due to survey dropout, crude counts become progressively lower across the survey and counts will not sum to the total sample of the 10,541 who initially completed the questionnaire for Sex Now. Estimation model inputs are presented in **Table 1**.

**Table 1:** Canadian provincial gay, bisexual, and other men who have sex with men population size estimation model inputs

Data source	Model input	n	%
range	range
CCHS	Gay/bisexual identifying participants, among sample	496,594	3.5
		449,009–544,179	3.1–3.8
	Self-reported p12m anal sex with a man, gay/bisexual participants, among sample	218,705	1.5
		184,565–252,845	1.3–1.8
	Self-reported p12m anal sex with a man, participants identifying as another sexual identity^a^, among sample	21,380^b^	0.1^b^
		12,146–30,613^b^	less than 0.1–0.2^b^
Sex Now^c^	Gay/bisexual identifying participants, among sample	9,693	92.0
		9,639–9,747	91.4–92.5
	Likely to disclose gay/bisexual identity on a government survey, among gay/bisexual participants	8,241	86.5
		8,175–8,307	85.8–87.2
	Self-reported p6m anal sex with a man, among gay/bisexual participants	4,561	78.8
		4,511–4,611	77.9–79.6
	Self-reported p6m anal sex with a man, participants identifying as gay/bisexual, among sample	4,561	94.9
		4,511–4,591	94.3–95.5
Statistics Canada	Canadian provincial 15 years of age and older male population count estimate	15,762,949	N/A
		N/A	N/A

Abbreviations: CCHS, Canadian Community Health Survey; N/A, not applicable; p6m, past 6 months; p12m, past 12 months

^a^ Participants who do not identify as gay or bisexual

^b^ Estimates are associated with a moderate amount of sampling variability (coefficient of variation: 15.0<CV<35.0), and caution in interpreting these data is warranted

^c^ Progressive respondent dropout/non-response and questionnaire skip logic have been accounted for in calculation of Sex Now proportions

The national Identity-or-Behaviour gbMSM population size in 2020 was estimated at 669,613 (4.3% of the male aged 15 years and older population). Among these, an estimated 39,310 (0.2% of the male aged 15 years and older population) did not identify as GB and reported anal sex with a man in the past 1–5 years. The estimate of gbMSM reporting past 6–12 months anal sex with a man, the Behaviour-only estimate was 412,186, representing 2.6% of the male aged 15 years and older population in Canadian provinces and 61.6% of the above Identity-or-Behaviour estimate. The Behaviour-only estimate includes 25,129 (0.2% of the male aged 15 years and older population) men who did not identity as GB but reported past 6–12 months anal sex with a man (**Table 2**).

**Table 2:** Canadian provincial gay, bisexual, and other men who have sex with men population size estimates, 2020

gbMSM definition	Population size estimate	% of male aged 15 years and older population
	range	range
Identity-or-Behaviour	669,613^a^	4.3^a^
	653,781^a^–685,446	4.2^a^–4.4
Behaviour-only	412,186^a^	2.6^a^

301,070^a^–523,301

1.9^a^–3.3

Abbreviation: gbMSM, gay, bisexual and other men who have sex with men

^a^ Estimates are associated with a moderate amount of sampling variability (coefficient of variation: 15.0<CV<35.0), and caution in interpreting these data is warranted

By province, British Columbia (4.6% of the male aged 15 years and older population) was found to have the greatest proportion of gbMSM based on the Identity-or-Behaviour definition, with the Atlantic regions (3.3% of the male aged 15 years and older population) found to have the lowest. Using the Behaviour-only definition, Ontario had the largest proportion of gbMSM among the male aged 15 years and older population (2.9%) and the Atlantic region had the lowest proportion (1.8%). Regardless of anal sex experience, a greater proportion of gbMSM reside in large population size areas than the national estimate, with around 10% of gbMSM in Canadian provinces residing in small population areas. Estimates stratified by region and by small and large population areas are presented in the Appendix.

## Discussion

In Canadian provinces during 2020, the gbMSM population size was estimated at 669,613 (representing 4.3% of the male aged 15 years and older population) based on our Identity-or-Behaviour definition. Among this, 94.1% self-identified as GB—regardless of sexual experience—and the remaining 5.9% did not identify as GB and reported past 1–5 years anal sex with a man. The Behaviour-only based estimate was 412,168 (representing 2.6% of the male aged 15 years and older population). This estimate comprises 93.9% self-identified GB individuals who reported past 6–12 months anal sex with a man and 6.1% men who did not self-identify as GB but who reported past 6–12 months anal sex with a man. These population estimates are invaluable in precise estimation of HIV incidence, prevalence, HIV testing and HIV pre-exposure prophylaxis uptake rates among gbMSM, which, in turn, are useful in program planning and resource allocation ((2,3,20,21)). These estimates will also be useful for inference at the population-level to quantify biobehavioural surveillance indicators among MSM.

Canada’s previous national gbMSM estimate was published in 2014, producing a population size of 349,837 (representing 2.4% of the male aged 15 years and older population). This estimate was derived from a direct weighted measurement of men who self-identified as GB and reported any sex with a man in the past 12 months in the CCHS and Québec Population Health Survey ((8)). It should be noted that this estimate did not include MSM who do not self-identify as GB, nor did it include an adjustment for nondisclosure on a governmental survey. While not directly comparable, the authors believe this previous estimate was likely an underestimate. Comparing to the present study’s Identity-or-Behaviour estimate (669,613, 4.3% of the male aged 15 years and older population), the increase may be partially explained by the inclusion of groups previously unaccounted for. The literature base is sparse on analyses of gbMSM identity versus sexual behaviour, thus limiting comparison. However, recent analyses of CCHS data, the General Social Survey from the United States and polling suggest a growing proportion of North American populations identify as 2SLGBTQ+, particularly among younger age groups, despite same-sex behaviour increasing at a lower rate ((22–24)). This latter point may be reflected in this analysis, with the Behaviour-only estimate accounting for approximately 62% of the Identity-or-Behaviour estimate. It is important to note that, among a wide spectrum of sexual behaviours, these analyses account for only anal sex behaviour, as a risk for HIV transmission and other STBBIs. The Identity-or-Behaviour estimate may include unmeasured sexual behaviours other than anal sex, but as a limitation of the data this could not be determined. Incorporating more precise population size estimates in epidemiological modelling will lead to more accurate understanding of the HIV epidemic and comparison across jurisdictions and population groups.

While others have demonstrated success using WotC, multiplier method, successive/respondent-driven sampling and mapping estimation, lack of nationally representative data precluded such methods within this study. Further, WotC methods seem particularly suited to small-area estimation and tend to produce estimates on the lower range of plausibility ((9,10,25)).

## Limitations

Sex Now and CCHS data, due to the self-report nature of their collection, are subject to measurement error, recall bias and reporting bias. However, due to the community-based nature of Sex Now, the authors believe sexual orientation reporting bias within CCHS is mitigated by our adjustment for non-disclosure. Additionally, Salway *et al.* show that assumed bias in non-probabilistic samples of sexual minority individuals may be overstated ((26)).

The data sources that were used to arrive at these estimates deserves some attention. It was found that 44.0% of GB-identifying CCHS respondents reported anal sex with a man in the past 12 months. Among GB-identifying Sex Now respondents, 78.8% reported past six months anal sex with a man. It is reasonable to suspect the CCHS sample, because of its interviewer-administration, to be subject to a greater amount of social desirability bias. While in contrast the Sex Now sample, given its promotion methods, may recruit a more sex-positive sample. Indeed, this was the impetus for taking the midpoint between these estimates as the main finding, resulting in the Behaviour-only estimate representing 62% of the Identity-behaviour estimate.

Some of the results from CCHS, as a limitation of a small sample size, are subject to high rates of sampling variability. This was evident in the national estimate of anal sex experience among men who do not self-identify as GB. Further, high sampling variability was particularly apparent in analyses stratified by region and population size area. Despite this, the authors believe the results to be plausible and provide them for interested readers.

These estimates, which incorporate sexual behaviour, have been limited to anal sex. It is important to note that many other STBBIs are readily transmitted through other routes, including oral sex. This is a limitation of CCHS data, which defines “sex” as vaginal or anal sex. As such, caution against the application of these estimates to other infections that are not transmitted through anal sex is warranted. Expanding the definition of sex within the CCHS to include oral sex, or including questions on other sex behaviours in future cycles is encouraged and may allow for population size estimation for broader STBBI considerations.

A limitation of CCHS data is the lack of information on sexual behaviours from residents in the northern Territories of Canada, which precluded estimation for these areas. Including this module of questions in future CCHS cycles may allow for more complete regional estimates. A gender-based analysis was unable to be applied to the data. However, Sex Now estimates account for gender diversity among respondents who do not identify as a woman.

Finally, estimates stratified by age were not produced in this study. With plans to update these estimates periodically, the authors hope to provide further stratification, including by age group, in future analyses.

## Conclusion

Using data from multiple sources, models used to estimate the population size of gbMSM, accounting for community members previously not included in prior Canadian estimates have been described. Namely, models account for GB-identifying individuals who would not be willing to disclose their sexual orientation on a population health survey and men that do not identify as GB but report anal sex with a man. The Identity-or-Behaviour and Behaviour-only estimates allow data users and policymakers to apply the estimate that best fits their needs. Specifically the Identity-or-Behaviour estimate, which relates directly to community size, while the Behaviour-only estimate may be more relevant for HIV and other STBBIs transmitted through anal sex.

## Appendix

**Table A1:** Canadian provincial gay, bisexual, and other men who have sex with men population size estimation model inputs, by region

Data source	Model input	Region	n	%
range	range
CCHS	Gay/bisexual identifying participants, among respective regional samples	Atlantic	24,690	2.7
			17,765–31,616	1.9–3.4
		Québec	122,155	3.7
			98,894–145,377	3.0–4.4
		Ontario	192,792	3.5
			160,889–224,694	2.9–4.0
		Prairies	81,754	3.1
			61,243–102,265	2.4–3.9
		British Columbia	75,243	3.9
			58,096–92,390	3.0–4.7
	Self-reported p12m anal sex with a man, gay/bisexual participants, among respective regional samples	Atlantic	7,411^a^	0.8^a^
			2,887–11,936^a^	0.3–1.3^a^
		Québec	37,986^a^	1.1^a^
			22,901–53,070^a^	0.1–1.6^a^
		Ontario	100,841	1.8
			78,072–123,609	1.4–2.2
		Prairies	42,766^a^	1.6^a^
			25,877–59,655^a^	1.0–2.3^a^
		British Columbia	29,701^a^	1.5^a^
			19,017–40,386^a^	1.0–2.1^a^
	Self-reported p12m anal sex with a man, participants identifying as another sexual identity^c^, among respective regional samples	Atlantic	1,120^b^	0.1^b^
			0–2,502^b^	0.0–0.3^b^
		Québec	3,013^b^	0.1^b^
			0–6,247^b^	0.0–0.2^b^
		Ontario	13,435^a^	0.2^a^
			5,433–21,437^a^	0.1–0.4^a^
		Prairies	2,137^b^	0.1^b^
			163–4,111^b^	0.1–0.2^b^
		British Columbia	1,675^b^	0.1^b^
			72–3,278^b^	<0.0–0.2^b^
Sex Now^d^	Gay/bisexual identifying participants, among respective regional samples	Atlantic	802	90.0
			784–820	88.0–92.0
		Québec	2,199	92.9
			2,178–2,225	92.0–94.0
		Ontario	2,969	92.2
			2,939–3,000	91.3–93.2
		Prairies	1,984	90.8
			1,957–2,011	89.6–92.1
		British Columbia	1,793	92.5
			1,716–1,762	91.3–93.7
	Likely to disclose gay/bisexual identity on a government survey, among gay/bisexual participants	Atlantic	680	86.5
			661–699	84.1–88.9
		Québec	1,962	90.8
			1,936–1,988	89.6–92.0
		Ontario	2,454	84.1
			2,416–2,492	82.8–85.4
		Prairies	1,643	83.7
			1,612–1,674	82.1–85.3
		British Columbia	1,502	88.5
			1,476–1,527	87.0–90.0
	Self-reported p6m anal sex with a man, among gay/bisexual participants	Atlantic	330	74.3
			315–345	70.9–77.8
		Québec	1,029	80.9
			1,006–1,052	79.0–82.7
		Ontario	1,367	77.3
			1,339–1,395	75.7–78.9
		Prairies	967	78.6
			945–989	76.8–80.4
		British Columbia	868	80.7
			847–889	78.8–82.6
	Self-reported p6m anal sex with a man, participants identifying as gay/bisexual, among sample	Atlantic	330	92.7
			320–340	90.0–95.4
		Québec	1,029	95.6
			1,016–1,042	94.4–96.9
		Ontario	1,367	94.2
			1,349–1,384	92.9–95.4
		Prairies	967	95.2
			954–980	93.9–96.5
		British Columbia	868	95.7
			856–880	94.4–97.0
Statistics Canada	Canadian Provincial male aged 15 years and older population count estimate	Atlantic	1,029,995	N/A
			N/A	N/A
		Québec	3,596,333	N/A
			N/A	N/A
		Ontario	6,119,281	N/A
			N/A	N/A
		Prairies	2,835,632	N/A
			N/A	N/A
		British Columbia	2,181,708	N/A
			N/A	N/A

Abbreviations: CCHS, Canadian Community Health Survey; N/A, not applicable; p6m, past 6 months; p12m, past 12 months

^a^ Estimates are associated with a moderate amount of sampling variability (coefficient of variation: 15.0<CV<35.0), and caution in interpreting these data is warranted

^b^ Estimates are associated with a large amount of sampling variability (coefficient of variation: ≤35.0) and do not meet Statistics Canada’s quality standards. Extreme caution in interpreting these data is warranted

^c^ Participants who do not identify as gay or bisexual

^d^ Progressive respondent dropout/non-response and questionnaire skip logic have been accounted for in calculation of Sex Now proportions

**Table A2:** Canadian provincial gay, bisexual, and other men who have sex with men population size estimates, by region, 2020

gbMSM definition	Region	Population size estimate	% of male aged 15 years and older population
		range	range
Identity-or-Behaviour	Atlantic	34,403^a^	3.3^a^
		33,255^a^–35,550	3.2^a^–3.5
	Québec	152,309^a^	4.2^a^
		148,390^a^–156,227	4.1^a^–4.3
	Ontario	270,604^b^	4.4^b^
		267,371^b^–273,837	4.4^b^–4.5
	Prairies	112,927^a^	4.0^a^
		108,728^a^–117,126	3.8^a^–4.1
	British Columbia	100,143^a^	4.6^a^
		97,217^a^–103,069	4.5^a^–4.7
Behaviour-only	Atlantic	18,259^a^	1.8^a^
		10,861^a^–25,657	1.1^a^–2.5
	Québec	85,590^a^	2.4^a^
		48,399^a^–122,781	1.4^a^–3.4
	Ontario	177,168^b^	2.6^b^
		146,910^b^–207,427	2.4^b^–3.3
	Prairies	72,937^a^	2.6^a^
		57,986^a^–70,797	2.0^a^–3.1
	British Columbia	59,938^a^	2.8^a^
		39,012^a^–80,364	1.8^a^–3.7

Abbreviation: gbMSM, gay, bisexual and other men who have sex with men

^a^ Estimates are associated with a large amount of sampling variability (coefficient of variation: ≤35.0) and do not meet Statistics Canada’s quality standards. Extreme caution in interpreting these data is warranted

^b^ Estimates are associated with a moderate amount of sampling variability (coefficient of variation: 15.0<CV<35.0), and caution in interpreting these data is warranted

**Table A3:** Canadian provincial gay, bisexual, and other men who have sex with men population size estimation model inputs, by area population size

Data source	Model input	Area population size	n	%
			range	range
CCHS	Gay/bisexual identifying participants, among sample	Large population area	418,516	3.3
			373,197–463,835	2.9–3.7
		Small population area	78,078	2.6
			64,063–92,092	2.1–3.0
	Self-reported p12m anal sex with a man, gay/bisexual participants, among sample	Large population area	189,522	1.5
			156,929–222,115	1.2–1.7
		Small population area	29,183^a^	0.9^a^
			18,587–39,779^a^	0.6–1.3^a^
	Self-reported p12m anal sex with a man, participants identifying as another sexual identity^b^, among sample	Large population area	16,517^a^	0.1^a^
			7,832–25,202^a^	0.3–0.8^a^
		Small population area	4,863^a^	0.2^a^
			1,914–7,811^a^	0.1–0.3^a^
Sex Now^c^	Gay/bisexual identifying participants, among sample	Large population area	7,042	92.6
			6,995–7,086	92.0–93.2
		Small population area	1,326	91.0
			1,304–1,348	89.1–92.1
	Likely to disclose gay/bisexual identity, among gay/bisexual participants	Large population area	6,181	89.3
			5,958–6,235	88.6–90.1
		Small population area	1,054	81.0
			1,026–1,082	78.8–83.1
	Self-reported p6m anal sex with a man, among gay/bisexual participants	Large population area	3,947	79.9
			3,902–3,992	79.0–80.8
		Small population area	618	72.5
			597–639	70.1–75.0
	Self-reported p6m anal sex with a man, participants identifying as gay/bisexual, among sample	Large population area	3,947	95.3
			3,920–3,974	94.6–95.9
		Small population area	618	93.1
			605–631	91.1–95.0
Statistics Canada	Canadian Provincial male aged 15 years and older population count estimate	Large population area	12,703,406	N/A
			N/A	N/A
		Small population area	3,059,543	N/A
			N/A	N/A

Abbreviations: CCHS, Canadian Community Health Survey; N/A, not applicable; p6m, past 6 months; p12m, past 12 months

^a^ Estimates are associated with a moderate amount of sampling variability (coefficient of variation: 15.0<CV<35.0), and caution in interpreting these data is warranted

^b^ Participants who do not identify as gay or bisexual

^c^ Progressive respondent dropout/non-response and questionnaire skip logic have been accounted for in calculation of Sex Now proportions

**Table A4:** Canadian provincial gay, bisexual, and other men who have sex with men population size estimates, 2020

gbMSM definition	Area population size	Population size estimate	% of male aged 15 years and older population
		range	range
Identity-or-Behaviour	Large population area	618,482^a^	4.9^a^
		605,494^a^–631,470	4.8^a^–5.0
	Small population area	76,101^a^	2.5^a^
		74,205^a^–77,996	2.4^a^–2.6
Behaviour-only	Large population area	388,008^a^	3.1^a^
		285,475^a^–490,542	2.3^a^–3.9
	Small population area	42,511^a^	1.4^a^
		29,966^a^–55,056	1.0^a^–1.8

Abbreviation: gbMSM, gay, bisexual, and other men who have sex with men

^a^ Estimates are associated with a moderate amount of sampling variability (coefficient of variation: 15.0<CV<35.0), and caution in interpreting these data is warranted
